# A systematic approach to the development and validation of adaptive manikins

**DOI:** 10.1186/2046-7648-4-S1-A15

**Published:** 2015-09-14

**Authors:** Agnes Psikuta, Manuela Weibel, Rick Burke, Mark Hepokoski, Tony Schwenn, Simon Annaheim, René M Rossi

**Affiliations:** 1 Laboratory for Protection and Physiology, Empa, St. Gallen, Switzerland; 2 Measurement Technology Northwest (MTNW), USA; 3 ThermoAnalytics, USA

## Introduction

Recent advances in computation technologies have facilitated computer simulation of human physiological regulation mechanisms at high spatial and temporal resolution. Improvements in manufacturing techniques and control strategies have resulted in the development of advanced thermal manikins. However, the broader acceptance of human thermophysiological simulation via modelling and measurement tools is limited by the scarce public domain resources and availability of validation data supporting such tools [1]. In this study a systematic approach to the development and validation of thermophysiology models and adaptive manikins was developed. This approach is based on both the evaluation of manikin responsiveness - to be able to follow the course of human physiological responses, and the adequate validation of an adaptive manikin against human experiments representing groups with increasing complexity of exposure.

## Methods

The responsiveness of the manikin Newton (MTNW, USA) was evaluated by addressing its accuracy (uncertainty of the heat flux measurement and lateral heat flow between segments) and dynamic control (response of manikin control system to the frequent change of the set-point temperature and manikin passive thermal behaviour beyond the limits of the control system). Secondly, a systematic approach to the validation of adaptive manikin (manikin Newton controlled by Manikin PC^2 ^physiology model (ThermoAnalytics, USA)) was based on selection of human experiments representing groups with increasing complexity of exposure (basic exposures with a wide range of steady-state and transient environmental conditions, low activity level and no clothing, active exposures with added variety of activity levels, and complete exposures considering clothing under spatial and temporal transient conditions).

## Results and discussion

The manikin responsiveness evaluation revealed the opportunities and constraints of this highly sophisticated thermal manikin, such as good measurement accuracy even with heterogeneous surface temperature, sufficiently fast response during passive heating and cooling. The dynamic regulation of surface temperature under extreme transient conditions was deficient; however, this parameter was not crucial in the adaptive manikin operation principle. This information was used for targeted selection of validation exposures in the subsequent validation study, as well as for better understanding of discrepancies between human and adaptive manikin data.

Secondly, the manikin controlled by the physiology model was successfully validated against fifteen selected human subject trials including all three exposure groups and original clothing used in human studies. The overall root-mean-square deviations for skin and core temperatures measured in humans and simulated using the adaptive manikin approximated 1.25 °C and 0.26 °C, respectively. These values were comparable with the typical inter-subject variability observed in human studies.

## Conclusion

In general, the manikin was suitable for coupling with a physiology model with respect to its accuracy and dynamics required for realistic simulation of the physiological response. The adaptive manikin was challenged in the validation with highly transient environmental conditions and long duration of the exposure, and it showed good performance in simulating the overall human thermophysiological response for both steady-state and transient conditions.
